# FunSwin: A deep learning method to analysis diabetic retinopathy grade and macular edema risk based on fundus images

**DOI:** 10.3389/fphys.2022.961386

**Published:** 2022-07-25

**Authors:** Zhaomin Yao, Yizhe Yuan, Zhenning Shi, Wenxin Mao, Gancheng Zhu, Guoxu Zhang, Zhiguo Wang

**Affiliations:** ^1^ College of Medicine and Biological Information Engineering, Northeastern University, Shenyang, Liaoning, China; ^2^ Department of Nuclear Medicine, General Hospital of Northern Theater Command, Shenyang, Liaoning, China; ^3^ College of Computer Science and Technology, Key Laboratory of Symbolic Computation and Knowledge Engineering of Ministry of Education, Jilin University, Changchun, Jilin, China

**Keywords:** fundus image, diabetic retinopathy, macular edema, disease stage prediction, swin transformer

## Abstract

Diabetic retinopathy (DR) and age-related macular degeneration (AMD) are forms of degenerative retinal disorders that may result in vision impairment or even permanent blindness. Early detection of these conditions is essential to maintaining a patient’s quality of life. The fundus photography technique is non-invasive, safe, and rapid way of assessing the function of the retina. It is widely used as a diagnostic tool for patients who suffer from fundus-related diseases. Using fundus images to analyze these two diseases is a challenging exercise, since there are rarely obvious features in the images during the incipient stages of the disease. In order to deal with these issues, we have proposed a deep learning method called FunSwin. The Swin Transformer constitutes the main framework for this method. Additionally, due to the characteristics of medical images, such as their small number and relatively fixed structure, transfer learning strategy that are able to increase the low-level characteristics of the model as well as data enhancement strategy to balance the data are integrated. Experiments have demonstrated that the proposed method outperforms other state-of-the-art approaches in both binary and multiclass classification tasks on the benchmark dataset.

## 1 Introduction

An eyeball is an impressively ingenious structure, with an optical system that mimics a traditional camera, and the fundus which functions as the photographic plate of the camera, allows one to see the dynamic of the blood circulation and the health status of the human body (Musadiq et al., 2003; Lee and Szema, 2005). For example, various characteristics of certain complications of diabetes, hypertension, coronary heart disease, and kidney disease can be identified in the fundus (Nathan, 1993; Wong et al., 2001; Grunwald et al., 2010; Xu et al., 2021). Presently, fundus photography is a commonly used method for screening the fundus. This technique enables visual perception of structure, which allows us to determine if there is any abnormality in the fundus (Yannuzzi et al., 2004).

Diabetic retinopathy (DR) and age-related macular degeneration (AMD) are two ophthalmic diseases that can be diagnosed through fundus photographs. Basic clinical manifestations of DR will appear on fundus images as neovascularization, capillary hemangiomas, vasodilation, hemorrhage, and occlusion of capillaries and arterioles (Stitt et al., 2016), whereas the basic manifestations of AMD will appear on fundus images as mainly the alteration of fundus macula (Zarbin et al., 2014). Unfortunately, in the early stages of the disease there may not be obvious clinical symptoms evident in the fundus image, making diagnosis challenging (Agurto et al., 2011).

Deep learning has made great strides in medical image diagnosis over the last decade. In particular, a number of deep neural networks have been modified and applied for detecting diseases related with fundus images in recent years. For example, several structural features of biological damage, such as blood vessels, fundus hemorrhage, and exudate, are added to advanced neural networks to train classification models based on artificially designed features (Alqudah et al., 2018; Ghani et al., 2019; Dong et al., 2021). These neural frameworks can also be trained using simple image characteristics such as pixel intensities (Das et al., 2021; Kanimozhi et al., 2021; Selçuk et al., 2022). Besides focusing on feature innovations, scientists will also focus on methodological innovations, such as developing a high performance deep neural network and integrating different machine learning algorithms with ensemble models (Sikder et al., 2021; Du et al., 2022).

It is true that these state-of-the-art methods have provided good results, however, many of them do not offer a diagnosis of disease staging, at the same time, for the detection of the above two diseases, they must be enhanced. Furthermore, the diagnostic performance of the models needs to be enhanced for the above two diseases. To address the above two pivotal questions, we propose a deep learning method based on Swin transformer. The main contributions of this work are summarized as follows:

For one thing, the appropriate benchmark model and other modules are selected and optimized for integrating a suitable deep learning framework for analyzing fundus images in accordance with the specific research objectives. For another, a series of highly reliable preprocessing operations are implemented based on the properties of the fundus images, while ensuring the integrity of the distribution of the data, thereby enhancing the accuracy of the resulting prediction. Finally, the ImageNet-based transfer learning mode is used as the basis training model in order to obtain sufficient low-level features for the learning. Consequently, when the model is fused with high-dimensional features, the model can be perceived more clearly (particularly some potential disease classification bases) and classification accuracy can be improved.

The paper is organized as follows: In Section 2, we provide an overview of the datasets and a brief description of the methods. In Section 3, we present experimental results and conclusions based on these results. Finally, in Section 4, we conclude the paper with a brief summary.

## 2 Materials and methods

**FIGURE 1:**
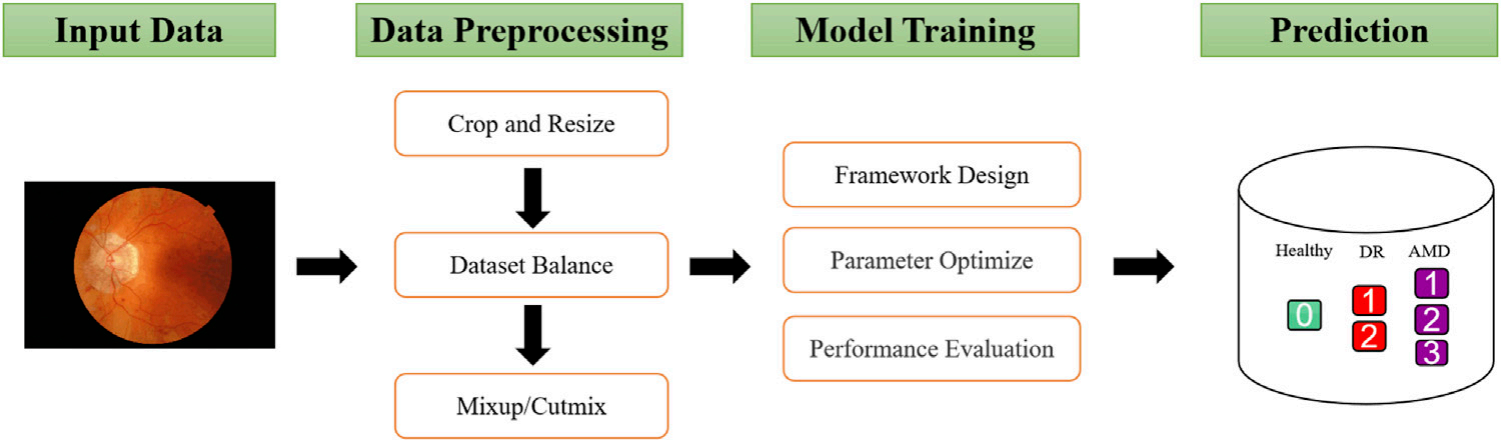
Overview of the proposed methodology.

As shown in Figure 1, This study involved four major stages: Dataset curation, data preprocessing, model training and prediction. The collected fundus images are first cut into squares and normalized to the same size, then the samples are balanced based on the number of each class. In addition, mixup and cutmix are used to further process the data. Since the Swin Transformer demonstrates excellent performance on other medical image classification problems, this framework is used in this study, and its parameters are adjusted based on the model’s performance. Finally, the binanry and mutil classification performance of the optimized will be evaluated by the evaluation metics. As a reminder, “Binary Classification” refers to the classification of health and disease, whereas “Multiclassification” refers to the classification of health and disease at different levels. The details of each process are described in the following sections.

### 2.1 Benchmark dataset

In this article, we used data from the MESSIDOR dataset (Decencière et al., 2014) that is available online for public use. There are 1,200 color numerical images of the posterior pole of the eye in this dataset, which correspond to two diseases: Diabetic retinopathy (4 levels, 0–3) and macular edema (3 levels, 0–2), where level 0 corresponds to a healthy subject. Medical experts have provided diagnoses for each image. Full details are available in Supplementary Tables S1, S2.

### 2.2 Data preprocessing

Firstly, only the middle part of the image is intercepted, which contains all pixels within the field of view, in order to lessen the interference caused by large areas of black background. And the size of these small crops is set to 960×960 . As a second step, the simplest data enhancement methods (rotation and mirroring) are used to increase the number of minority samples, eliminating the effects of categories imbalance and maintaining an equal distribution of the dataset. For example, As regards diabetic retinopathy, the sample sizes for 0–3 levels are 546, 153, 247, and 254 respectively. On level 1 samples, we use 180-degree rotation, mirroring, mirroring+180-degree rotation to expand them to three times their original size. While level 2 and 3 samples are expanded by 180-degree rotation and mirroring. This procedure allows each subclass to attain an approximate balance in the number of samples and eliminates the impact of imbalanced categories. Lastly, the internal mix-up and cut-mix methods of the network (Liu et al., 2021) will also be used to optimize the performance of the Swin Transformer. CutMix and MixUp enable us to create inter-class examples. CutMix randomly interpolates the pixel values between two images and places fragments of one image over another, while MixUp randomly interpolates the pixels between two images. The two processes prevent the model from being overfitted to the training distribution and improve its likelihood of being able to generalize to examples outside of the distribution. A further benefit of CutMix is that it prevents a model from over relying on any particular feature when it is performing its classifications.

### 2.3 Model training

#### 2.3.1 The pipeline of the framework

The deep learning framework implemented in this study consists of three components, which are the backbone, the neck, and the head.

**FIGURE 2:**
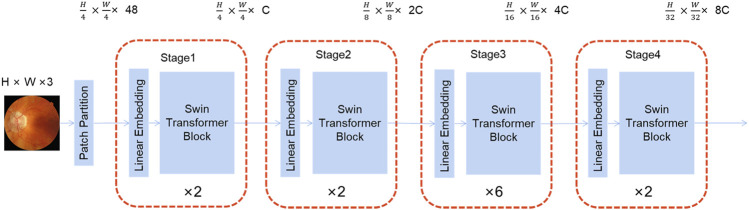
The framework of the Swin transformer.

As illustrated in Figure 2, the Swin Transformer is served as the framework’s backbone. Its structure is reminiscent of a convolutional hierarchy and the resolution is reduced by half, while the number of channels is doubled. The first patch partition divides the image into a series of blocks, followed by four stages, each of which contains two parts: patch merging (the first block is linear) and Swin Transformer block. Patch merging is similar to pooling; however, it does not lose information in the process.

**FIGURE 3:**
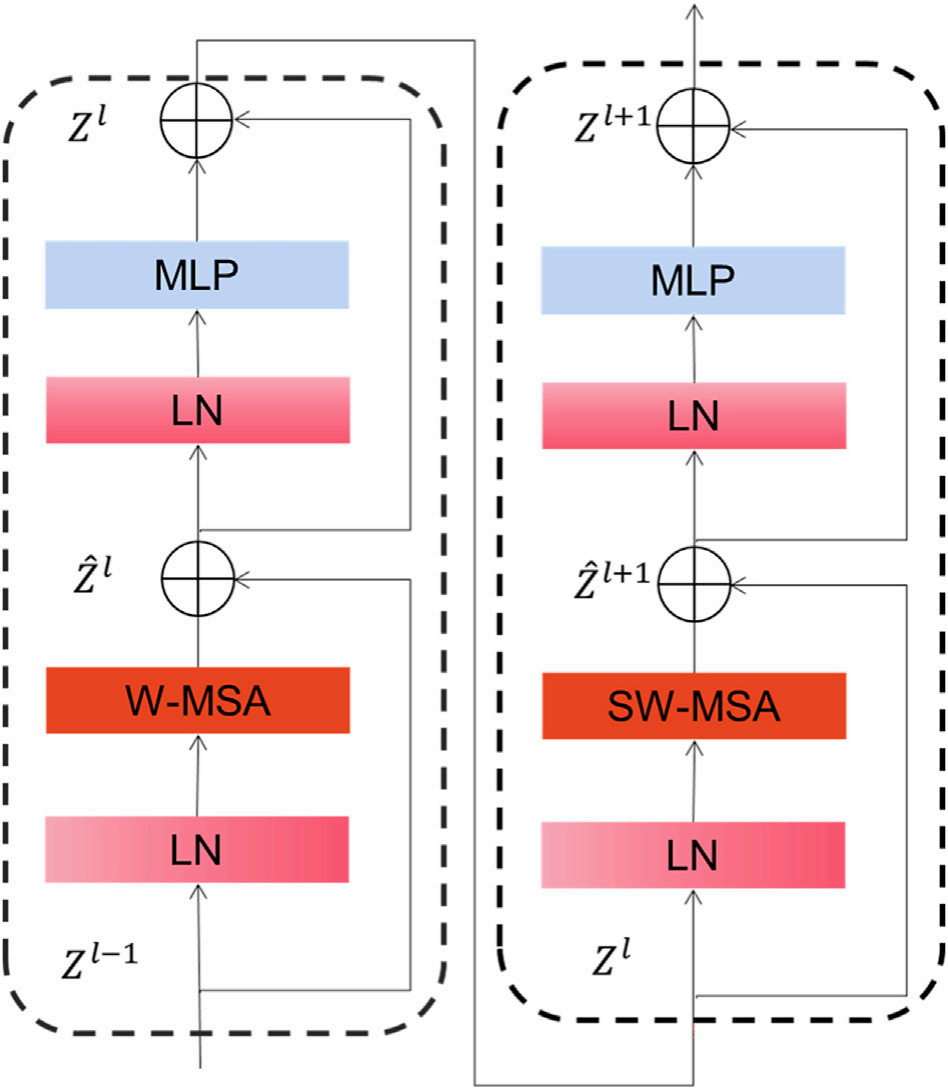
The details of the Swin Transformer Block.

As depicted in Figure 3, Swin Transformer Block is basically similar to a common transformer block except that it uses window multi-head self-attention (W-MSA) and mobile window multi-head self-attention (SW-MSA) to replace multi-head self-attention (MSA) module. With this moving-window method, self-focused computations are limited to a non-overlapping local window, allowing for inter-window connectivity. Moreover, this hierarchical converter is capable of modeling images of various sizes and has linear computation complexity. As a result of these features, Swin converter is highly competitive in handling a wide variety of visual tasks (Zhang et al., 2021; Jiang et al., 2022).

Global Average Pooling (GAP) composes the neck of the framework. There are several advantages of GAP over traditional fully ensemble layers. One is that it is more suitable for convolutional structures by improving the compatibility of function maps and categories, another is that there are no parameters to adjust in the global media collection, meaning that overestimation at the global level can be reduced. It contributes to the achievement of good results in many network structures for medical data (Bien et al., 2018; Valan et al., 2019; Shahhosseini et al., 2021).

Linear CLS is the head of the framework. Using this module makes the model relatively simple and easier to train since the mapping between features and categories is clearly visible. Its loss function is described as follows:

$$Loss=-\overset{n}{\underset{i=1}{\sum }}y\left(i\right)log\left(p\left(x_{i}\right)\right)$$ (1)

Where $p\left(x_{i}\right)$ is the result of the model output computed by softmax and y(i) is calculated as follows:

$$y\left(i\right)=\{\begin{array}{cc} \frac{\alpha }{n}, & i\neq class\\ 1-\alpha +\frac{\alpha }{n}, & i=class \end{array}$$ (2)

Where n is the number of categories, i is the predicted label, and class is the current real category. Where α is smoothing coefficient, which we set to 0.1, as in (Liu et al., 2021).

#### 2.3.2 Parameter setting

This framework has the following parameters: the batch size is set to 32, the epoch is set to 600, and the initialization policy of the CLS head is TruncNormal, which has a standard deviation of 0.02. Additionally, AdamW is used as the optimizer with a learning rate of 0.0001 and decay rate of 0.05. All other parameters are set to the default values.

#### 2.3.3 Performance metrics

Performance metrics have been employed to assess the predictive performance of our models, including sensitivity, specificity, accuracy, and F1-score. In medical image analysis, these evaluation measurements are well established and they have been used in the benchmark studies on the diagnosis of fundus-related diseases as well. The metrics are calculated as follows.

$$\{\begin{array}{c} Acc=\frac{TP+TN}{TP+FP+TN+FN}\\ SN=RE=\frac{TP}{TP+FN}\\ SP=\frac{TN}{TN+FP}\\ PR=\;\frac{TP}{TP+FP}\\ F1-Score=\;\frac{2\;\times \;PR\;\times RE}{\;PR+RE} \end{array}$$ (3)

The variables TP, FP, TN and FN represent the true positive, false positive, true negative, and false negative values, respectively. RE and PR represent recall and precision.

## 3 Results and discussions

### 3.1 The result of binary classification on diabetic retinopathy and macular edema

This paper compares nine state-of-the-art methods that have been widely used in medical imaging in recent years, which are Conformer (Peng et al., 2021), ConvNeXt (Liu et al., 2022), HRnet (Wang et al., 2020), Vgg 11 (Simonyan and Zisserman, 2014), Mlp-Mixer (Tolstikhin et al., 2021), Res2Net 50 (Gao et al., 2019), ShuffleNet V1 (Zhang et al., 2018), T2T Vit (Yuan et al., 2021) and Vit Transformer (Dosovitskiy et al., 2020).

As depicted in Table 1, the proposed method yielded the best results for diabetic retinopathy on both the accuracy and F1- score, and for sensitivity, our effect is second only to that of VIT Transformer, which is only 0.43% worse. With regard to specificity, our effect ranks third, which is 10.36% less than that of the highest-performing model. Similarly, for macular edema, as illustrated in Table 2, accuracy, F1-score, and sensitivity of the proposed model have reached the highest values, 98.66, 98.96, and 98.68%, respectively, while their specificity is 1% lower than the best model.

**TABLE 1:** Comparing the existing methods of binary classification for diabetic retinopathy.

Methods	Accuracy	Sensitivity	Specificity	F1-score
Conformer	0.8855	0.8817	0.8963	0.9193
Convnext	0.8267	**0.9613**	0.4451	0.8913
HRnet	0.8378	0.8796	0.7195	0.8891
Vgg11	0.8156	0.8538	0.7073	0.8725
Mlp Mixer	0.8585	0.9226	0.6768	0.9060
Res2net50	0.8267	0.8452	0.7744	0.8782
Shufflenet_v1	0.8045	0.8796	0.5915	0.8693
T2T Vit	0.8553	0.8323	**0.9207**	0.8948
Vit Transformer	0.8076	0.9419	0.4268	0.8786
Our Method	**0.9062**	0.9376	0.8171	**0.9366**

**TABLE 2:** Comparing the existing methods of binary classification for macular edema.

Methods	Accuracy	Sensitivity	Specificity	F1-score
Conformer	0.9745	0.9755	0.9727	0.9801
Convnext	0.9320	0.8962	**0.9966**	0.9443
HRnet	0.9453	0.9283	0.9761	0.9563
Vgg11	0.7947	0.7283	0.9147	0.8204
Mlp Mixer	0.9441	0.9453	0.9420	0.9561
Res2net50	0.9648	0.9566	0.9795	0.9722
Shufflenet_v1	0.9648	0.9698	0.9556	0.9726
T2T Vit	0.9587	0.9377	**0.9966**	0.9669
Vit Transformer	0.9611	0.9604	0.9625	0.9695
Our Method	**0.9866**	**0.9868**	0.9863	**0.9896**

The relationship between specificity and sensitivity is often asymmetric, so it is very challenging to make sure both will produce positive results. We have maintained that the objective of this project is to better screen out patients with diseases, therefore in terms of method design and model training, we have sought a higher degree of sensitivity. Perhaps this explains why our model is less specific than other models in binary diagnostics of these disorders.

### 3.2 The results of multi-classification on diabetic retinopathy and macular edema

Having to deal with the problem of multi-classification, macro-average is used to calculate these indicators. According to this principle, increasing the proportion of each category of images will increase the weight of that category. The final result of the indicator is the sum of the results obtained from multiplying the corresponding indicator results of each subcategory by their respective weights.

Table 3 and Table 4 illustrate that the proposed method is superior to other models in various indicators with regard to the multiclassification problem of diabetic retinopathy and macular edema. In comparison with the binary classification problem, our model does not demonstrate any reduction in the multi-classification problem. It may be that there are only three subcategories to classify, or that there are distinct features which separate subcategories.

**TABLE 3:** Comparing the existing methods of multi-classification for diabetic retinopathy.

Methods	Accuracy	Sensitivity	Specificity	F1-score
Conformer	0.7704	0.7524	0.9122	0.7691
Convnext	0.7123	0.6754	0.8770	0.6999
HRnet	0.7374	0.7031	0.8954	0.7311
Vgg11	0.6211	0.5630	0.8547	0.6013
Mlp Mixer	0.7248	0.7121	0.8942	0.7246
Res2net50	0.6352	0.5568	0.8665	0.6054
Shufflenet_v1	0.6101	0.6500	0.8383	0.6026
T2T Vit	0.6950	0.7023	0.8679	0.6845
Vit Transformer	0.7563	0.7268	0.9150	0.7490
Our Method	**0.8412**	**0.8154**	**0.9413**	**0.8400**

**TABLE 4:** Comparing the existing methods of multi-classification for macular edema.

Methods	Accuracy	Sensitivity	Specificity	F1-score
Conformer	0.9733	0.9733	0.9865	0.9733
Convnext	0.8214	0.8147	0.9104	0.8091
HRnet	0.8761	0.8725	0.9379	0.8745
Vgg11	0.6136	0.6035	0.8056	0.5753
Mlp Mixer	0.9210	0.9202	0.9602	0.9209
Res2net50	0.8882	0.8849	0.9443	0.8872
Shufflenet_v1	0.9635	0.9638	0.9816	0.9636
T2T Vit	0.9174	0.9144	0.9583	0.9160
Vit Transformer	0.9514	0.9510	0.9754	0.9514
Our Method	**0.9866**	**0.9866**	**0.9932**	**0.9866**

### 3.3 Performance of data enhancement on the model

The data augmentation test was conducted on the binary classification. Table 5 summarizes the changes to evaluation indicators of the proposed method before and after data enhancement. Following data enhancement, almost all indicators were improved. There are two main factors contributing to this: The first is the expansion of minorities by rotating and flipping against class imbalances; this allows the data to be more balanced, reduces the impact of unbalanced data on the model, and enhances its performance. Additionally, fundus images the physiological structure reflected by fundus images is relatively fixed, that is, the distribution of segmentation targets in fundus images is essentially regular, and the semantic understanding of these targets is rather straightforward. So, low-resolution information provides specific features that are necessary for target object recognition. Although the model has gained sufficient low-level features from migration learning, there are still only a limited number of original images available for input, which means that the enhanced images compensate for the lack of original data.

**TABLE 5:** Performance of binary classification before and after data augmentation.

	Methods	Accuracy	Sensitivity	Specificity	F1-score
Diabetic Retinopathy	No Augmentation	0.5444	1.0	0	0.7050
	Augmentation	0.9062	0.9376	0.8171	0.9366
Macular Edema	No Augmentation	0.9389	0.7941	0.9726	0.8308
	Augmentation	0.9866	0.9868	0.9863	0.9896

### 3.4 Performance of transfer learning on the model

An assessment of transfer learning was conducted on the binary classification. Accordingly, the accuracy, sensitivity, specificity, and F1-score values of diabetic retinopathy are respectively 0.7393, 1, 0 and 0.8501. Similarly, these indicators of macular edema are 0.6457, 1, 0, and 0.7847. ACC and F1 of both diseases increased significantly following the addition of transfer learning.

### 3.5 Convergence of the model

**FIGURE 4:**
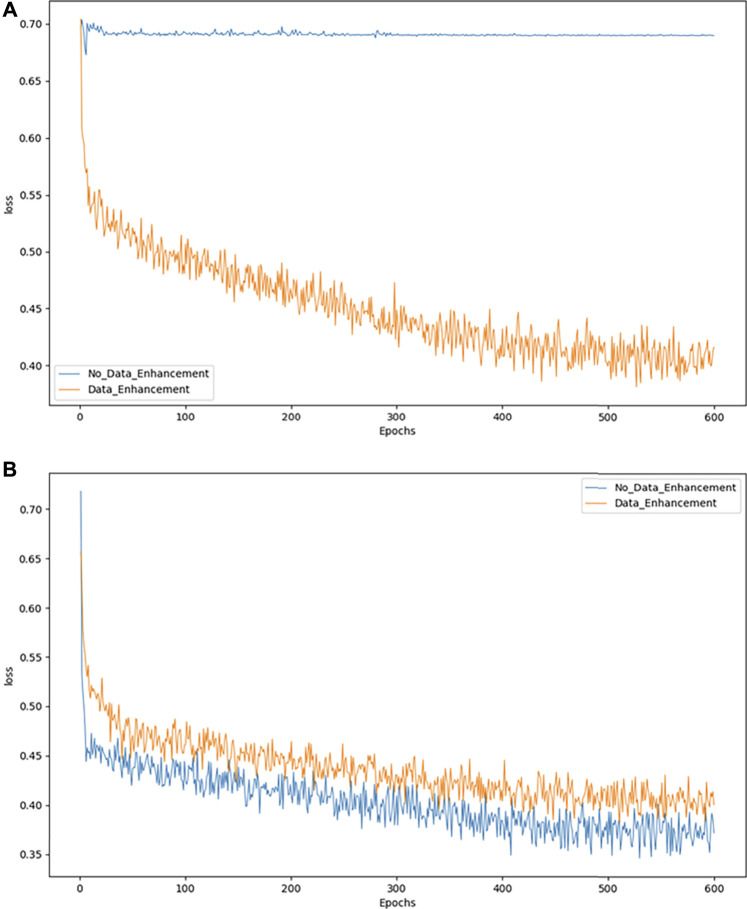
Model convergence performance of binary classification.

As an example, we exploit the binary classification problem to demonstrate the effects of model convergence. Figures 4A,B show the convergence of the model in DR and MD respectively. The abscissa represents the number of epochs used, while the ordinate represents the value of the loss suffered by the epoch. As shown in Figure 4A, the model converges more effectively only after data augmentation. While in 4B the model without data augmentation achieves better convergence, it is not evident from the actual test results.

Loss declines may not be apparent when the model cannot solve the problem of category imbalance. Accordingly, we can consider balancing the data set to resolve this issue. However, when the number of pictures increases because of a data balance, the overall loss may increase slightly, although this may be due to the increase in images, which does not adversely affect our classification accuracy.

### 3.6 Running environment and time cost

The experiments were conducted using a computing server with an Intel i9-11900K CPU, an NVIDIA RTX-3090 GPU, and Kingston 32 GB memory. Ubuntu 18.04 is the operating system of the server. In general, the training time for a picture is 0.02 s and the testing time is 0.007 s.

## 4 Conclusion

In this project, a method referred to as FunSwin is proposed as a means to solve the problem of grading diabetic retinopathy and estimating macular edema risk using fundus images. The basic framework for the method is Swin Transformer, with some modules based on some features of medical data to improve performance. In comparison to the existing studies on this benchmark dataset, FunSwin was found to outperform the existing studies in binary classifications and multi-classifications of these two diseases. Furthermore, as regards binary classification, when each subcategory of disease is given the same amount of training data, i.e., assuming that all data for each subcategory is balanced, then the binary classification effect of the model will still be improved. The study however, may need further evaluations in the clinical practices. There have been very few clinical studies on AI-based retinal diseases due to a variety of challenges, such as regulatory requirements and the annotations of experienced clinicians. Additionally, there is no specific evidence that these fundus-related symptoms are directly connected to particular diseases. After receiving ethical approval and the accumulating of a large, well-annotated dataset, this limitation of the proposed method will be resolved in a future study. In future studies, we also intend to use FunSwin in treating other retinal disorders, such as stroke, heart disease, etc.

## Data Availability

Publicly available datasets were analyzed in this study. The data can be found at: https://www.adcis.net/en/third-party/messidor/[MESSIDOR DATASET].
