# Supplementary material for: FunSwin: A deep learning method to analysis diabetic retinopathy grade and macular edema risk based on fundus images
Source: Front Physiol. 2022 Jul 25;13:961386. doi: 10.3389/fphys.2022.961386 (PMC9358036; doi:10.3389/fphys.2022.961386)
Supplement: Supplementary file 1 [file Table1.DOCX]

**Supplementary Table S1. Details of MESSIDOR dataset** (The classification of a disease is typically based on its class value, and the higher the value, the more serious the disease. The class 0 refer to healthy samples)

| **Diseases** | **Classes** | **Numbers** |
| --- | --- | --- |
| Retinopathy grade | 0 | 546 |
|  | 1 | 153 |
|  | 2 | 247 |
|  | 3 | 254 |
| Macular Edema | 0 | 974 |
|  | 1 | 75 |
|  | 2 | 151 |
