# Supplementary material for: FunSwin: A deep learning method to analysis diabetic retinopathy grade and macular edema risk based on fundus images
Source: Front Physiol. 2022 Jul 25;13:961386. doi: 10.3389/fphys.2022.961386 (PMC9358036; doi:10.3389/fphys.2022.961386)
Supplement: Supplementary file 2 [file Table2.docx]

**Supplementary Table S2. Sample Images of MESSIDOR dataset**

| **Class** | **Grade** | **Sample Image** |
| --- | --- | --- |
| Healthy | 0 | 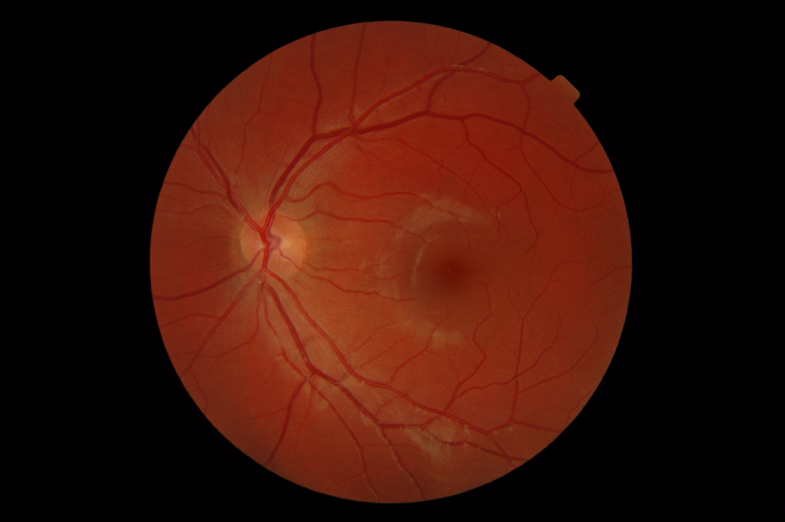 |
| Retinopathy grade | 1 | 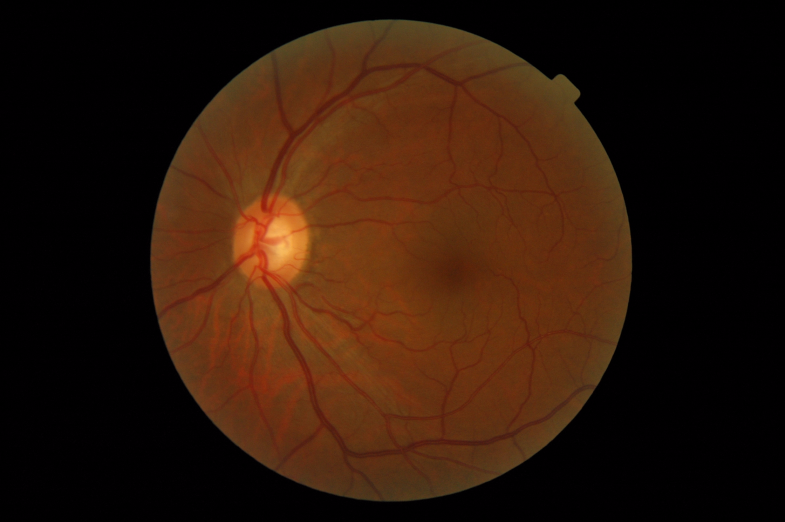 |
|  | 2 | 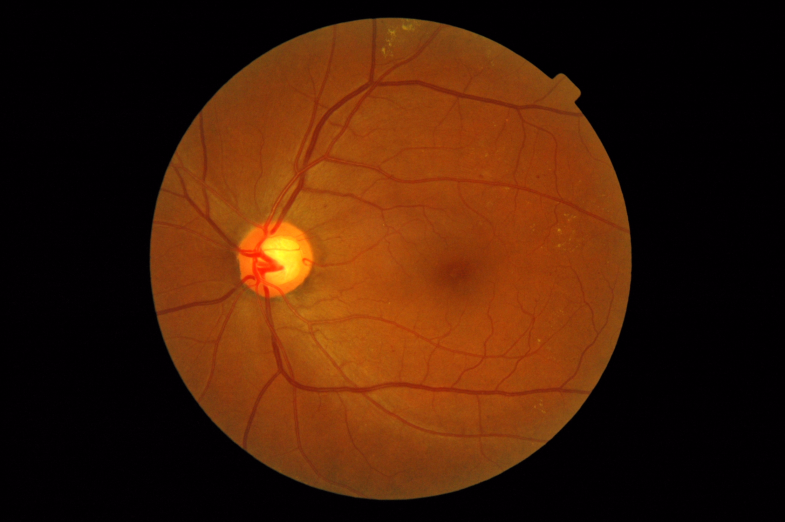 |
|  | 3 | 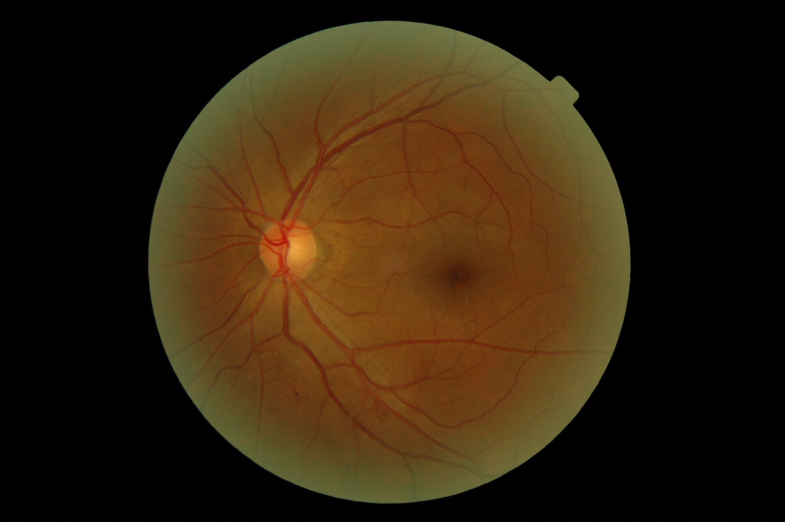 |
| Macular Edema | 1 | 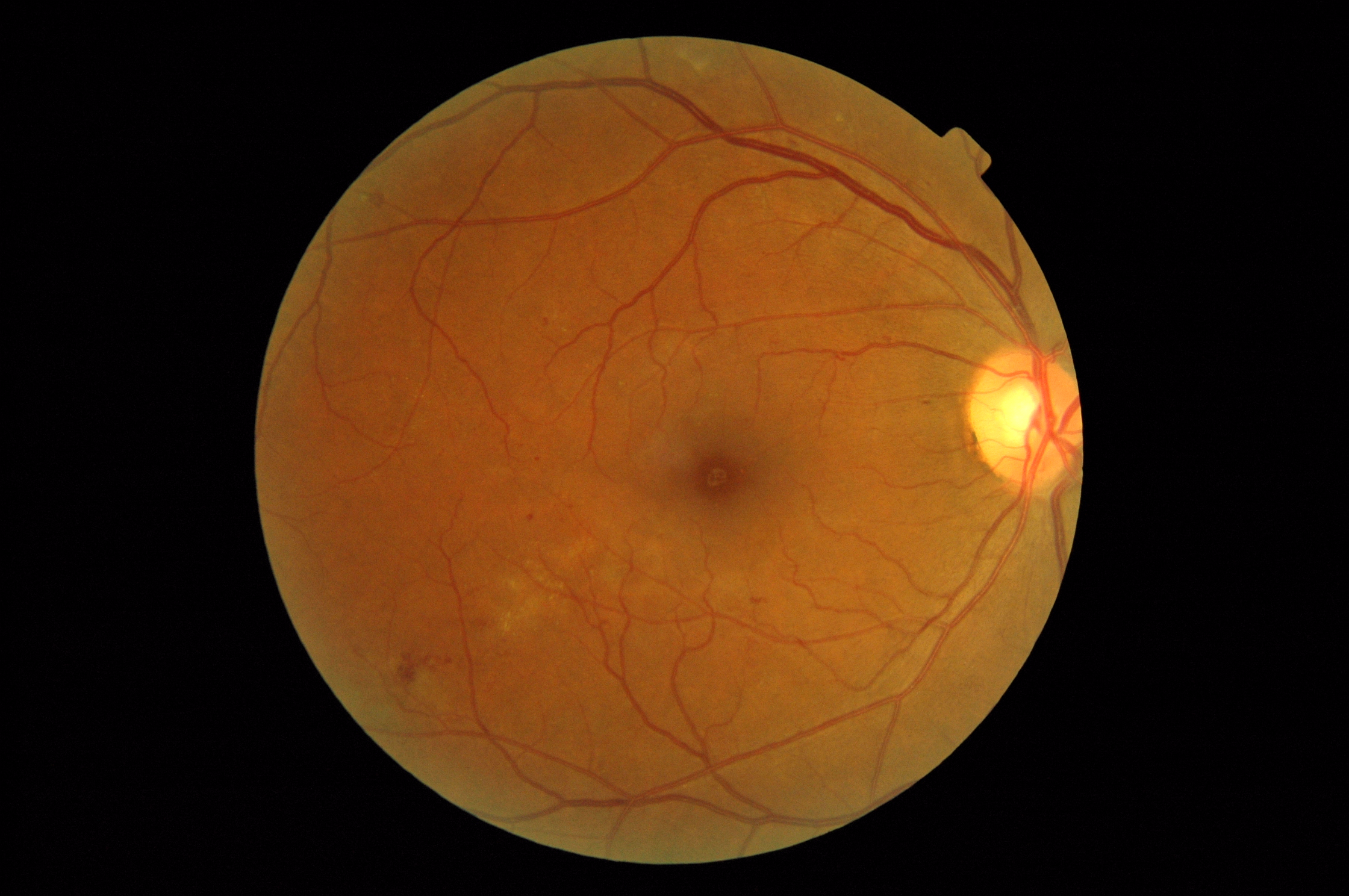 |
|  | 2 | 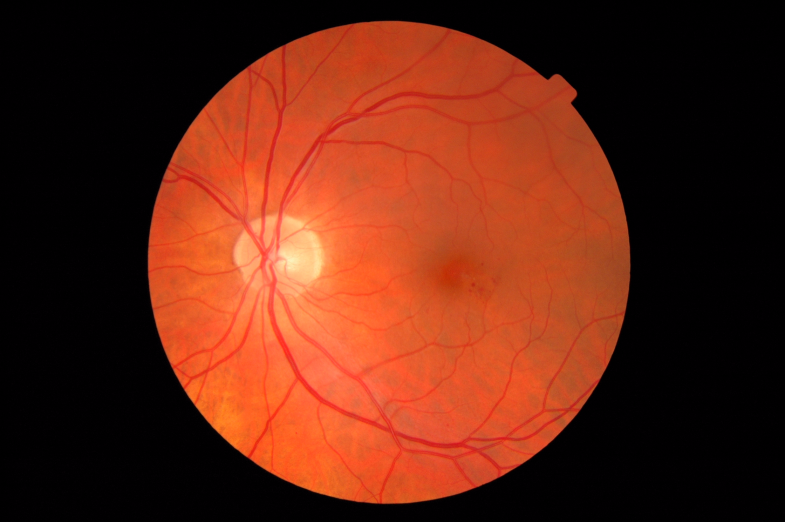 |
